# Reply to correspondence on “The importance of data transformation in correlation analysis of FVIII and inhibitor titers in acquired hemophilia”

**DOI:** 10.1016/j.rpth.2026.103345

**Published:** 2026-01-20

**Authors:** Dandan Yu, Wei Liu, Lei Zhang

**Affiliations:** Institute of Hematology & Blood Diseases Hospital, Chinese Academy of Medical Sciences & Peking Union Medical College, Tianjin, China

To the Editor:

We have read with great interest the letter to the editor [1] entitled “The importance of data transformation in correlation analysis of FVIII and inhibitor titers in acquired hemophilia,” which raises important methodological considerations regarding our recent study on acquired hemophilia A [2]. We sincerely thank the correspondent for their attention to our work and for initiating this insightful discussion on a key statistical issue.

We fully concur that the application of data transformation techniques, such as logarithmic transformation, represents a more robust strategy for uncovering potential associations when analyzing typically skewed variables like factor (F)VIII activity and inhibitor titers. To evaluate this proposition directly, we performed a reanalysis of our dataset.

**Figure 1:**
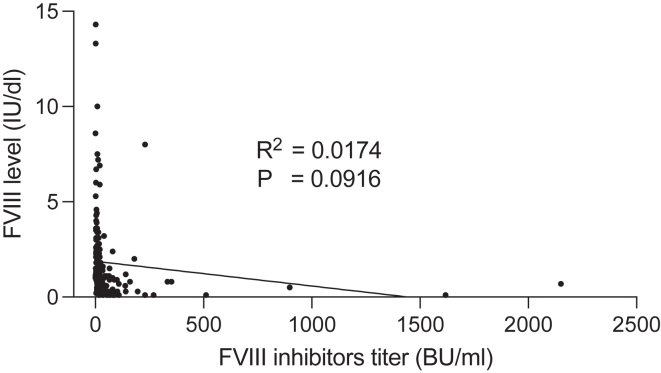
Simple regression of factor (F)VIII level vs FVIII inhibitor titer (raw data).

**Figure 2:**
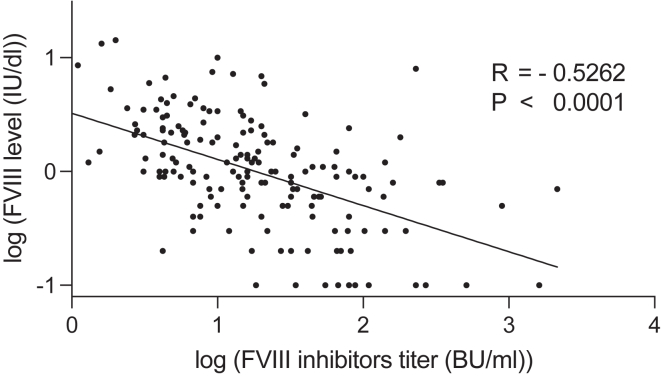
Simple regression of log(factor [F]VIII level) vs log(FVIII inhibitor titer).

In close agreement with the findings presented in the letter, our results demonstrated the following: analysis of the raw data showed no significant correlation between FVIII activity and inhibitor titers (*r*^2^ = 0.0174; *P* = .0916) (Figure 1). Following logarithmic transformation of both variables, the analysis revealed a moderate but highly significant negative correlation (*r* = –0.5262; *P* <.0001) (Figure 2). Furthermore, the transformed data exhibited a linear relationship with more symmetric distributions, thus rendering them a more suitable foundation for correlation analysis than the original, highly skewed data.

We also performed an additional analysis using Spearman correlation on the raw data. This nonparametric method is robust to skewed distributions and nonlinear relationships. The results confirmed a significant negative correlation (ρ = −0.5457; *P* < .0001), which aligns with the Pearson correlation analysis on the log-transformed data.

We wish to take this opportunity to clarify a methodological discrepancy in our original article 2]. The Methods section incorrectly cited the use of Spearman correlation. In fact, the analysis initially performed and reported was a Pearson correlation applied directly to the raw, nontransformed data, which led to the erroneous conclusion of no significant association. We sincerely apologize for this oversight.

These results strongly support the correspondent’s central thesis: neglecting appropriate data transformation can indeed obscure legitimate statistical associations between variables. We are grateful that the correspondence prompted us to identify this significant relationship, which was masked by the skewness of the raw data. Based on this experience, we recommend that when analyzing markedly skewed variables, a robust analytical approach is to use both Spearman correlation (on the raw data) and Pearson correlation (on transformed data) as complementary methods. This dual strategy not only leverages the robustness of the nonparametric test but also explores the potential for a linearized relationship, thereby providing a more comprehensive and reliable assessment of association.

We extend our renewed appreciation to the correspondent for their valuable input. This constructive exchange underscores the critical importance of rigorous methodological consideration in biomedical research. We are committed to incorporating robust analytical strategies, including data transformation, in our future studies, and we encourage fellow researchers in the field to similarly evaluate these approaches in their analyses of analogous data.
